# Conflict of interest prevention clause in the constitution: The study of the Indonesian Constitution

**DOI:** 10.1016/j.heliyon.2023.e14679

**Published:** 2023-03-20

**Authors:** Ibnu Sina Chandranegara, Dwi Putri Cahyawati

**Affiliations:** University of Muhammadiyah Jakarta, Indonesia

**Keywords:** Conflict of interest, Constitution, Separation of power

## Abstract

This study starts from the fact that Indonesia has adopted the separation of powers after reforming the state administration system. However, the separation of powers after twenty years was only formally against state power. Meanwhile, absolute power is not separate. The question is about the influence and involvement of economic power in state administrators. For example, the Indonesian law-making process for the Mining Law of 2020 and the Job Creation Law of 2020 was hijacked by political-business interests, which were biased between business and public interests. Many state administrators are affiliated with entrepreneurs, resulting in conflicts of interest in law-making and decision-making. This study assumes that a clause on preventing conflicts of interest must be formulated in the Constitution as the highest law of the land and state ethics. Therefore, this study aims to answer why the Constitution must include the conflict of interest clause. Also, how the substance of the prevention conflict of interest clause. The normative research method is used in this study by conducting a historical and comparative analysis of clauses to prevent conflicts of interest. This study also formulated ideal clauses to determine what actions are considered to create conflicts of interest that potentially impact law-making and decision-making.

## 1 Introduction

After the 1945 Indonesian Constitution amendment in 1999–2002, the presidential system was transplanted from the United States government system [[1], [2], [3]]. Adopting the American presidential approach has impacted Indonesia's constitutional system, such as the principles, mechanisms, and values embedded in the American presidential system. The main impact is the application of the principle of separation of powers, checks and balances mechanism. This formal separation of powers has significantly impacted the separation of powers between legislative, executive and judiciary [4,5]. However, such a formal separation cannot separate the firm relationship between the rulers in a formal sense and the real sources of power [6,7]. This condition then has the effect of creating a sharp gap in state practice. Moreover, the interaction between power in real terms often experiences conflicts of interest, such as law-making and decision-making, which is difficult to avoid. The conflict of interest is how the relationship between rulers and entrepreneurs is merged [8,9]. Therefore, it is assumed to encourage the decline of democracy [10].

Moore concludes that a large and independent class and urban population have become indispensable elements in the development of democracy [11]. Democracy will grow and develop if the bourgeoisie becomes solid and active in democratization; no bourgeoisie, no democracy [11]. Today many entrepreneurs agree with Moore's doctrine, and it is even marked by the number of entrepreneurs flocking to the world of politics and entering as state administrators. It is a growing trend, with many entrepreneurs holding public office [12,13].

During the New Order era, the role of entrepreneurs was only limited to a mere supporting system from political and economic networks. It was because the government faced the problem of a lack of capital. The government incentivised private capital owners or entrepreneurs in trust to cooperate with the government [12,14]. At that time, capital, contracts, concessions, and credit from the state were given directly to entrepreneurs, but the private entrepreneurs ran or used them on the same occasion [15,16]. They are regulated under the bureaucratic apparatus and are usually highly dependent on foreign capital. Therefore they are only supporting players behind the government [14]. However, this opportunity has entered the political sphere with comprehensive openness nowadays. In some shows, nominations can be bought to run for the legislature with powerful financial allure [17].

After the collapse of the New Order, the post-Soeharto political and economic constellation spread power everywhere, and its political influence was vast. As a result, efforts to get convenience and political protection in doing business are getting broader and more expensive. More and more entrepreneurs are approaching the centre of power by bribing, thus creating transaction costs for rent-seeking gains in power [18]. In addition, implementing a multiparty system - at the same time using a presidential system - after the amendment to the 1945 Constitution opened up extensive opportunities for entrepreneurs to get involved and participate in the world of politics [4,18,19]. This multiparty system requires each party to support itself. The financial strength of the party becomes one of the determinants of the party's strength to compete for power in the parliament, which in turn has an impact on bargaining for the placement of party people in parliament. One of the accesses to financial strength is obtained from entrepreneurs.

Consequently, a political party system like this gave birth to a patron-client relationship. Aspinall defines a patron-client relationship or patronage as distributing material resources for a specific purpose and providing political benefits. In particular, material resources are distributed through clientelistic networks based on personal power relations [20]. Hutchcroft defines clientelism as personal power relations of higher social status (patrons) with those of lower social status (clients) in mutual bonds [21]. However, over the last decade or so, a second wave of clientelism studies has emerged. Nowadays, it is uncommon for academics to emphasize aspects of hierarchy, dyarchy, and inequality in their conceptions of clientelism. Patron-client relationships have been unequal in recent decades due to democratic and economic modernization processes, but they are still widely prevalent. As a result, clientelism is now referred to as a specific kind of exchange rather than a relationship between patrons and clients. Most academics today define political clientelism as the act of exchanging a targeted, contingent, non-policy-based distribution of material rewards (cash, jobs, government contracts, services, legislation, etc.) for political support (such as votes, campaign funding and other forms of campaign support) [22]. In this paper, we use that definition. By using this definition, it can be said that a transaction involving a supporter of a politician is clientelistic if the supporter receives a benefit in a way that is not based on the impersonal application of a public policy and is instead thought to be dependent on the support the supporter provides to the politician. The above conditions will give birth to a situation that can cause a conflict of interest between state officials. Conflicts of interest will give birth to state administration which is “managed” by some parties (oligarchy), and in fact, the rule of law masks the true sources of power in society [[23], [24], [25]].

Ultimately, this will lead to regulatory capture or where the power of capital can “hostage” the laws and regulations to side with business interests rather than public interests [26]. For example, the controversy of regulatory capture and conflict of interest in the Indonesian law-making process on the Mining Law of 2020 and the Job Creation Law of 2020. The Mining Law of 2020 is accused of providing benefits to several companies whose licenses are expired. Meanwhile, some ministers are companies' shareholders, so it was carried out hastily and deemed not to comply with the carry-over mechanism [27]. On the other hand, the Job Creation Law of 2020 uses the first omnibus method in Indonesia, while the omnibus method and technique are not regulated in the Law-Making Law of 2011. This method is intended to protect large investors' interests due to the changing of 79 other sector laws to make business easier [28].

Based on this case, the law-making process was hijacked by political-business interests which were biased between business interests and public interests [27]. This tendency shows that a mere formal separation of powers is in fact difficult to create a limited government. This condition also explains that in substance a separation between business and political interests is necessary. Therefore, the absence of a conflict of interest prevention clause is a missing part that needs to be completed. So, This article intends to answer two questions, why is it essential to include a prevention clause in the 1945 Constitution, and how is the clause formulated in the 1945 Constitution?

The current paper followed the logical sequence of notions’ appearance. Part 1, as the Introduction section presents the Indonesian condition after constitutional reform, political and economic constellation spread power everywhere. As a result, efforts to get convenience and political protection in doing business are getting broader and more expensive. In return, voters provide political support, and entrepreneurs offer financial support to politicians. This situation creates a conflict of interest and impacts the law-making process. Part 2, the Method and material section, analyses the methodology used to conduct normative legal research on constitutional doctrines on conflict of interest, how it relates to the separation of power theory and the history of the constitutional provision on conflict of interest. Part 3, as the result of the research, will contain three subtitle analyses, among others (a) why is it essential to include the prevention of conflict of interest clause in the Constitution; (b) the conflict of interest clause in the Indonesian legal system; and (c) how is the conflict of interest prevention clause formulated. Finally, The paper closes with a brief conclusion in Part 4.

## 2 Methods and material

This study uses a normative legal research method. The normative legal research method is a process to find legal rules, principles and doctrines of the law to address the legal issues at hand [29]. The result of the study of law is the argument, theory, or new concept as a prescription for solving the legal problem [30]. This method, also known as legal research doctrinal, is research on laws drafted and developed based on the doctrine adopted by the developers [31]. So the object of study is the prevention of the conflict of interest clause that must be included in the Constitution, especially in Indonesia's Constitution. This research's legal materials include primary and secondary legal materials. The technique of collecting legal materials is carried out through literature studies. There are two variables analyzed. The first is the relationship between the separation of powers and preventing conflicts of interest clauses. Second, how the Indonesian constitutional system regulates conflicts of interest. Third, how the prevention of conflict of interest clause is formulated. Therefore, this study will use a historical and comparative approach. The materials for this study include the 1945 Constitution, the United Republic of Indonesia 1949 Constitution, the 1950 Constitution, the Government Administration Law and various ministerial regulations in government administration. In addition, this study conducted interviews with Constitutional Law experts. The interview result will present the genuine conception from the perspective of conflict of interest and the Constitution.

## 3 Result of research

### 3.1 Why is it essential to include the prevention of conflict of interest clause in the constitution?

Society is becoming more aware of the problems that conflicts of interest can cause. Conflicts of interest, for instance, could affect how accounting companies audit financial statements, how judges rule in court, and how doctors treat patients [32]. Conflicts of interest problems play a significant and well-known role in many fields of law [33]. For instance, ethical norms restrict attorneys' capacity to represent both sides of a dispute, corporation law principles forbid company directors from acting on insider knowledge, and agency law regulations forbid trustees from mixing their own finances with those of the trust. Conflicts of interest can be extremely important in forming constitutional doctrine [34,35]. However, this position is gravely undervalued [36]. Courts and academics occasionally bring up conflict issues when studying the Constitution. However, very few authors discuss the importance of conflicts of interest for constitutional interpretation, and those that do typically focus on a specific doctrine [32]. Consequently, constitutional law's treatment of or need for treatment of conflicts of interest has not been thoroughly explored by legal academia.

Avoiding conflicts of interest in the administration of the state is a fundamental part of public ethics [37]. Public ethics is a principle public officials must hold as state administrators [38]. In carrying out their duties as public servants, public officials must adhere to the principles of public ethics, where awareness in making certain decisions or policies must be based on noble values and the public interest. So it is because conflicts of interest can cause bias in the consideration/judgment of decisions [39]. A person has a conflict of interest if they are in a relationship with another, requiring them to exercise judgment in that other's service, and they have an interest tending to interfere with the proper exercise of judgment in that relationship [34,40]. Conflicts of interest can affect thought processes in two ways; conscious and subconscious (self-interest) [41]. So that such a situation will give birth to a moral dilemma; individuals tend to be more concerned with themselves than with common interests [42]. Ethical choices are not made in a closed space but are part of social interaction [43]. Ultimately, decisions are made based on social motives such as loyalty, maintaining trust, reciprocating or helping someone in a difficult situation [44]. There are at least three main types of conflicts of interest; *first,* an actual conflict of interest, where a conflict exists between your official duties or responsibilities and your private interests. *Second,* a perceived conflict of interest, where others could see that your interests could improperly interfere with or influence you in the performance of your official duties or responsibilities, whether or not this is, in fact, the case. *Third*, the potential conflict of interest, where your private interests could interfere with or influence your official duties or responsibilities in the future [43,44].

Based on the description above, it can be understood that conflicts of interest can arise when decision-makers represent many other people's interests, which conflict with each other. Conflicts can also occur when the decision-makers interests are at stake. For instance, a single intensive care unit bed may need to be assigned by a physician. However, another doctor's patient, who just so happens to be the first doctor's wife, needs intensive treatment at the same time. The first doctor thinks it is his duty as a professional to prioritize the needs of his patients, but in this situation, he is only able to do so for one patient, and that patient happens to be his family. Alternatively, imagine a lawyer helps a married couple prepare their inheritance and they decide to The attorney actively represents both clients. But if the husband and wife end up as clients of the advocate, they may not be able to completely discharge that role [32].

Although this kind of conflict is essential in many areas of law and other professions where a specific set of rules is laid down in a code of ethics, it is not a real concern in the Constitution. The doctrine of the Constitution, particularly regarding the separation of powers, accepts the idea that the President, legislators and judges will weigh and balance conflicting interests [32,45]. It is an essential part of the role of state officials to consider the needs of some constituents or parties with the requirements of other constituents or parties [46]. Furthermore, a doctrine explains the law-making motives known as a positive political doctrine. This doctrine assumes that political and bureaucratic actors are self-serving and behave strategically to advance their goals [47]. The focus is on the motives for the re-election of political actors in the future period and the desire of government officials for promotion and moonlighting in the private sector. This perspective is prominent in many scholars in explaining the behavioural motivations of legislative actors and executive agency actors in the law-making process [47].

The issue of conflict of interest can be expanded in the constitutional interpretation, particularly in exercising the powers granted by the Constitution [32]. The relationship between state powers bridged by the authorities becomes an additional problem if associated with a conflict of interest. Generally, a conflict of interest in the interpretation of the Constitution occurs when one of the state institutions extends its authority beyond the limits determined by the Constitution [48]. This expansion will generally reduce the authority of other institutions, ignore it, or even change the flow of the authorization process determined by the Constitution. Such conditions are typically reflected in the law-making process and judicial review [49].

### 3.2 The conflict of interest clause in the Indonesian legal system

In the Indonesian legal system, the regulation regarding the prevention of conflicts of interest is spread across various legal products. Still, it concentrates on government administrative procedures, especially in making decisions or actions. It is because decision-making often involves a conflict of interest, which affects the fulfilment of general principles of good governance [50]. So Article 42 to Article 45 Government Administration Law of 2014 stipulates, “The conflict of interest as referred to in Article 42 occurs if in determining and/or making decisions and/or actions are motivated by.

a. Personal and/or business interests;
b. Relationships with relatives and family;
c. Relations with representatives of the parties involved;
d. Relationships with those who work and receive salaries from the parties involved;
e. Relationship with the party providing recommendations to the parties involved; and/or
f. Relationships with other parties prohibited by the provisions of laws and regulations.

Article 42 of the Government Administration Law regulates the prohibition of making and/or making decisions and/or actions for government officials who have the potential to have a conflict of interest so that such decisions and/or actions must be determined and/or carried out by superiors or other officials. Violation of these provisions is threatened with light administrative sanctions. Likewise, officials who do not inform their superiors about potential conflicts of interest or superiors who do not immediately examine, research, and make decisions regarding the conflict of interest within 5 (five) days are threatened with light administrative sanctions. Meanwhile, Article 43 of the Government Administration Law regulates the criteria for a conflict of interest background, which results in a government official not being allowed to make and/or make decisions and/or actions. If there is an alleged conflict of interest, then Community Members have the right to report the allegation, as regulated in Article 44 of the Government Administration Law. Officials who make decisions and/or take actions that contain conflicts of interest and cause state losses are subject to heavy administrative sanctions.

The regulation of conflicts of interest at the level of the Government Administration Law provides clear guidelines for Government Officials to refrain from taking and/or stipulating actions and/or decisions. Although not all decision-making cases with a potential conflict of interest background lead to decisions contrary to the principles of government administration, normative restrictions are still needed to minimize the risk of abuse of authority while protecting Government Officials from mistakes.

Before issuing the Government Administration Law, this restriction was regulated in the Regulation of the Minister for Empowerment of State Apparatus and Bureaucratic Reform Number 37 of 2012 concerning General Guidelines for Handling Conflicts of Interest. The regulation mentions several forms of conflict of interest that often occur and are faced by state officials, among others.

1. A situation that causes a person to receive gratification or the giving/accepting of gifts for a decision/position;
2. A situation that causes the use of office/agency assets for personal/group interests;
3. Situations that cause confidential information of positions/agencies to be used for personal/group interests;
4. Concurrent positions in several agencies that have direct or indirect, similar or dissimilar relationships, thus causing the use of one position for the benefit of other positions;
5. A situation where a state administrator gives special access to certain parties, for example, in employee recruitment, without following proper procedures;
6. Situations that cause the supervisory process not to follow procedures due to the influence and expectations of the supervised party;
7. A situation where the authority to evaluate a qualifying object and the object is the result of the appraiser;
8. A situation where there is an opportunity for abuse of office;
9. Moonlighting or outside employment (work other than the main job); and
10. Specific situations that allow the use of discretion that abuses authority.

Apart from government administration procedures, the principle of *nemo judex idoneus in propria causa* is known in the justice field. This principle means that judges do not make decisions concerning their interests, either directly or indirectly. In other words, judges do not examine and decide or become judges in matters related to themselves [51], or simply that no one is a judge in their case [52]. This principle is a principle inherent in function. In this case, the Constitutional Court Justice is expected to solve the constitutional issue submitted to him. Therefore, impartiality is inherent and must be reflected in the stages of the case examination process up to the decision-making stage so that court decisions can truly be accepted as a fair legal solution for all litigants and the broader community in general [53].

This principle is close to the direction of impartiality of judges. Judges will base their decisions on the law and facts at trial, not based on their association with one of the litigants, nor will they decide the case themselves. Constitutional judges have impartiality regulated in Article 17 paragraph (5) of Judiciary Law states that “A judge or clerk is obliged to resign from the trial if he has a direct or indirect interest in the case being examined either at his will itself or the request of the litigants.” The elucidation states that what is meant by direct or indirect interest is if the judge or clerk, or other party has handled the case and has previously been related to the job or position concerned. This provision has also been revealed in Joint Decrees of the Supreme Court Chairman and the Judicial Commission No. 047/KMA/SKB/IV/2009 and No. 02/SKB/P.KY/IV/2009 and Regulation of the Constitutional Court Number 09/PMK/2006 Regarding the Enforcement of the Declaration of the Code of Ethics and Conduct of Constitutional Judges. The impartiality can only be done if the judge can escape from the conflict of interest or the spirit of friendship (collegial) with the litigants. Therefore the judge must resign from the trial if he sees the potential for impartiality. In the Indonesian legal system, judges must resign if they have a marital relationship with one of the litigants or are examined in court. Therefore, the judge must withdraw from the trial process if he sees the potential for impartiality [54].

### 3.3 How is the conflict of interest prevention clause formulated?

The clause on preventing conflicts of interest was regulated in Article 79 Paragraph (2) of the United Republic of Indonesia Constitution and Article 55 Provisional Constitution 1950, which states: [55].

(1) President and minister's positions may not be held together by holding any public office within and outside the Republic of the United States of Indonesia.
(2) The President and the Ministers may not, directly or indirectly, participate in or be a guarantor for any corporate entity based on a profit or profit agreement with the Republic of the United States of Indonesia or any part of Indonesia.
(3) They may not have debts at the expense of the Republic of the United States of Indonesia, except for general debentures.
(4) What is stipulated in paragraphs (2) and (3) of this article shall remain in effect for them for three years after resigning.

However, after issuing the Presidential Decree dated July 5, 1959, which reinstated the 1945 Constitution, such a clause was not re-applied. The loss of the conflict of interest clause harms the possibility of making decisions that are vulnerable to being influenced by their interests directly.

Business actors breaking through to power, either because of collusion or dual roles, have been included in the political economy study since the mid-1970s. In the end, he introduced the theory of economic rent-seeking [56]. This theory explains the phenomenon of entrepreneur behaviour to obtain special licenses, monopolies and other facilities from the authorities who have power over these fields. It is easy for other actors to enter the market with a special license [21,56]. Therefore, economic rent-seeking behaviour is usually anti-competitive behaviour or avoiding competition. A pile of empirical and comparative research shows that entrepreneurs, especially in developing countries, enter the circle of the power elite because entrepreneurs want to enjoy rent from the authorities by providing financial rewards and political support [57]. Empirical research in India, Pakistan, Malaysia, Thailand, and South Korea unravelled the intimate relationship between rulers and entrepreneurs pursuing economic rents to build business-politico groups [58,59]. Kunio called the capitalists developed in Southeast Asia entrepreneurs who grew up intimately connected with the regime. This quasi-entrepreneur builds a business by obtaining privileges and political protection [60]. In Thailand, Phongphaichit and Baker dissect the history of Shin Corporation's business development [61,62]. Thaksin Shinawatra, the former prime minister of Thailand, started a former police officer supplying computer equipment and stationery to police institutions. In the early 1990s, he got cable TV, telecommunications (paging, cell phone, card-phone), satellite and datanet concessions for 1.3 billion baht [63].

Phillips dissects the Bush Dynasty in the United States, built on the foundations of the financial, petroleum, and military industries. Prescott Bush, President Bush's grandfather, is a businessman-turned-senator from Connecticut and President Eisenhower's favourite golf buddy. From this business-political relationship, the Bush dynasty developed the most prominent oil entrepreneur in the United States [64]. In South Korea, the chaebols built their multinational corporations with the full support of the regime in power [65]. They have a cosy relationship with the authorities to obtain concessions and licenses. Chaebols seek rents not by investing their capital in industries that produce goods for profit, but they seek rents, in other words, by investing in an investment portfolio [66]. In the Philippines, entrepreneurs dominate the political and business spheres, constructed similarly, focusing on the President as the entrepreneur's patron. It was found that the state apparatus had all its components open to be plundered by powerful private sector forces. This system is known as booty capitalism [67].

The above phenomenon can illustrate how the characteristics of a purely profit-seeking business meet the interests of power. The size of the contribution of entrepreneurs can be seen as the size of the business interest in influencing the policies of political parties. These transactions usually occur while achieving power through elections and continue when power is acquired and implemented [68,69]. It impacts the ruling authorities of political parties in implementing policies, which the interests of entrepreneurs can easily influence as their support during elections. The distortion occurs when the business position should be outside the scope of power, reversing the reality. As a result, the role of power has changed, which should have a service character to become profit-oriented. It marks the death knell of democracy. It is related to complications due to the unification of three characters of political resources, namely entrepreneurs, businesses, and political parties.

The clause on preventing conflicts of interest makes sense, avoids conflicts, and binds the Constitution's basic principles, namely equality before the law. It is in line with what was stated by John Locke that “no man in civil society can be exempted from its laws” [70]. Including a conflict of interest prevention clause in the Constitution, at least avoiding a conflict of interest, helps ensure that government officials cannot exploit the government's position. They secure other institutional or personal benefits that are not their right. This clause will also encourage the adoption of a substantial separation of powers rather than just a separation of powers in a formal sense. Therefore, a conflict of interest prevention clause can be included by formulating it as formulated in the United Republic of Indonesia Constitution and the 1950 Constitution. State officials may not be in concurrent positions carrying out any public office inside and outside the Republic of Indonesia, and they may not be directly or indirectly in charge of a business entity that obtains a license. In addition, they may not have debts to the state and against the President and/or Vice President and regional heads; no person who has blood relations or conflicts of interest with the incumbent is not allowed to advance in the election process. As for what is meant by ‘not having a conflict of interest with the incumbent’ is not having blood relations, marital ties and/or lineage of 1 (one) level straight up, down, sideways with the incumbent, namely father, mother, in-laws, uncles, aunts, brother, sister, brother-in-law, child, son-in-law unless it has passed a gap of 1 (one) term of office.

## 4 Conclusion

Conflicts of interest should be given a significantly larger role in constitutional interpretation than they do at the moment, according to the findings of this study. The notion of separation of powers is based on the idea that disputes include the same fundamental concern about the abuse of political power. Therefore, taking into account conflicts naturally ties into fundamental constitutional ideas.

One would anticipate that considering disputes will offer crucial insights into constitutional interpretation, given its connection to a fundamental constitutional principle. The arguments in this article demonstrate how it can. Three important constitutional conundrums can be resolved by accounting for conflicts. First, it can reconcile the conflict between judicial supremacy and the political question doctrine, provide a sound functionalist theory for cases involving the separation of powers, and suggest when constitutional modification is appropriate.

Ultimately, the article emphasizes that preventing conflicts of interest deserves a much more significant role in the Constitution. This precautionary norm requires the same core concern about abuse of power that ultimately underlies the doctrine of separation of powers. So that the separation of powers is not interpreted as form and formal but also substantial. Therefore, this argument can round out the basic principles of constitutionalism, limitation of power, fulfilment of state ethics, and equality before the law.

Several clauses that prevent conflicts of interest in the Constitution include prohibiting state officials from having concurrent positions within and outside the country. They may not directly or indirectly be in charge of business entities, third may not have debts to the state, and there is a prohibition on dynastic politics.

## Author contribution statement

Ibnu Sina Chandranegara: Conceived and designed the experiments; Performed the experiments; Analyzed and interpreted the data; Contributed reagents, materials, analysis tools or data; Wrote the paper.

Dwi Putri Cahyawati: Analyzed and interpreted the data; Contributed reagents, materials, analysis tools or data.

## Funding statement

Ibnu Sina Chandranegara was supported by Universitas Muhammadiyah Jakarta [011/FHUMJ/I/2022].

## Data availability statement

Data included in article/supplementary material/referenced in article.

## Declaration of interest's statement

The authors declare that they have no known competing financial interests or personal relationships that could have appeared to influence the work reported in this paper.
